# DNA methyltransferases expressions in mice tongue exposed to waterpipe smoke

**DOI:** 10.1590/1678-7765-2025-0665

**Published:** 2026-02-16

**Authors:** Carolina Simão Flausino, Sarah Freygang Mendes Pilati, Filipe Ivan Daniel, Filipe Modolo

**Affiliations:** 1 Universidade Federal de Santa Catarina Programa de Pós-graduação em Odontologia Florianópolis Santa Catarina Brasil Universidade Federal de Santa Catarina, Programa de Pós-graduação em Odontologia, Florianópolis, Santa Catarina, Brasil.; 2 Universidade do Vale do Itajaí Departamento de Odontologia Itajaí Santa Catarina Brasil Universidade do Vale do Itajaí, Departamento de Odontologia, Itajaí, Santa Catarina, Brasil.; 3 Universidade Federal de Santa Catarina Departamento de Patologia Florianópolis Santa Catarina Brasil Universidade Federal de Santa Catarina, Departamento de Patologia, Florianópolis, Santa Catarina, Brasil.

**Keywords:** Waterpipe Smoking, Squamous Cell Carcinoma of Head and Neck, Epigenomics, DNA Methylation, DNA (Cytosine-5-)-Methyltransferase 1, DNA methyltransferase 3B

## Abstract

**Objective:**

Waterpipe smoking has gained widespread use nowadays, especially among young adults. As this type of smoke has been described to contain toxins and carcinogens, this study evaluated DNA methyltransferase (DNMT) 1 and 3b expression, and inflammation, on the dorsal surface, ventral surface and lateral border of Swiss mice’s tongues exposed to waterpipe smoke.

**Methodology:**

Animals were divided into 6 groups (n=60): control, 7, 15, 30, 60, and 90 days of consecutive exposure to waterpipe smoke in a whole-body exposure system. After each period, tongues were analyzed using hematoxylin/eosin staining for inflammation status and immunohistochemistry for DNMT1 and DNMT3b.

**Results:**

DNMT3b showed lower immunoexpression from 7 to 60 days; at 90 days, expression was similar to that of control or there was upregulation on the ventral surface compared with control. DNMT1 exhibited lower expression at all exposure times, with the ventral surface showing similar expression to that of control at 90 days. Waterpipe smoke was not able to induce acute or chronic inflammation in mice tongue.

**Conclusion:**

Waterpipe smoke may result in a DNA hypomethylation pattern in initial exposure periods, contributing to activate proto-oncogenes and/or genomic instability. Over long periods, it may lead to a methylation pattern similar to that of control or even to hypermethylation, silencing tumor suppressor genes. These alterations in the genome due to hypo/hypermethylation contribute largely for the development of diseases such as oral cancer.

## Introduction

The Middle Eastern culture has attained global prevalence and is propagating the practice of tobacco consumption via waterpipes.^1^ A waterpipe, also known as narghile, arghile, hookah, or shisha, is a tobacco smoking device that has gained increasing popularity, particularly among young adults, often supplanting conventional cigarette smoking among youth.^2,3^ It is well-documented that waterpipe smoke contains a plethora of toxic and carcinogenic substances derived from heated charcoal and flavored tobacco, including but not limited to nicotine, tar, polycyclic aromatic hydrocarbons, tobacco-specific nitrosamines, volatile aldehydes, benzene, nitric oxide, carbon monoxide, phenols, and heavy metals.^4,5^ The composition of these substances may vary depending on the waterpipe design and structure, duration of use, volume of water in the bowl, and even the porosity of the hose.^5,6^

Despite an inaccurate perception that waterpipe smoke is less harmful compared with conventional cigarettes,^3^ studies have elucidated that it contains higher amounts of toxins and carcinogens detrimental to the oral cavity and airway pathways compared to cigarette smoke.^7,8^ Consequently, its usage has become prevalent even in public spaces, particularly as a means of social inclusion among young individuals.^9,10^ Lack of specific legislation, varied research methodologies, and absence of the social stigma associated with conventional cigarettes have further fueled its widespread adoption.^11^

Oral squamous cell carcinoma (OSCC) ranks among the most prevalent malignancies worldwide, with approximately 335,000 new cases annually.^12^Tobacco use stands out as one of the primary risk factors influencing genetic and epigenetic alterations implicated in OSCC development.^13^ However, the association between oral cancer and waterpipe use remains unclear. Epigenetic changes such as modifications in DNA expression without alterations in the DNA sequence have garnered recent attention in OSCC research. Among these changes, DNA methylation, a heritable epigenetic modification catalyzed by DNA methyltransferases (DNMTs), has emerged as a significant contributor.^14^ DNMTs are enzymes responsible for catalyzing the covalent addition of a methyl group to the fifth carbon position of a cytosine within a guanine base in a CpG dinucleotide.^13^ While DNA methylation is crucial for normal development and survival, dysregulation of this process can lead to various diseases, including malignant neoplasms.^15^

DNMT1 is responsible for maintenance of DNA replication by copying the methylation pattern of the DNA mother strand to the daughter after each duplication.^16^ DNMT3b contributes to de novo methylation, a process that adds methyl groups to previously unmethylated DNA regions.^17^ Overexpression of DNMTs has been linked to OSCC development.^18^ DNMT1 overexpression is correlated with DNA aberrant methylation in solid tumors, lymph nodes metastasis, and poor prognosis for patients.^19^ Similarly, DNMT3b overexpression is associated with lymph node metastasis, higher recurrence rates, and poor prognosis.^17^

Given the global spread of waterpipe usage and its propensity to expose individuals to carcinogenic substances, this study assessed DNMT1 and DNMT3b immunoexpression and inflammation in the tongue of Swiss mice exposed to waterpipe smoke over varying time points.

## Methodology

### Animals and treatments

This study was approved by the Ethics Committee on Animal Use from Itajaí Valley University (approval number 063/17). Based on Martins, et al.^20^ (2012), sixty female three-week old Swiss mice were selected and maintained in conventional animal cages in an animal care unit at 24± 1ºC with a 12:12 light/dark cycle^20^. Water and pelleted food were available ad libitum.

### Waterpipe smoke exposure

**Figure 1:**
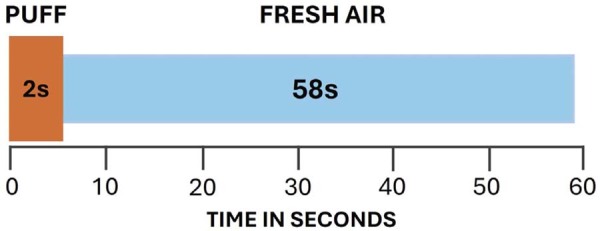
Schematic representation of one inhalation cycle used in the study.

Following a one-week acclimatization period, the mice were randomly assigned (n=10) to six groups: control (no exposure) and exposure to waterpipe smoke for 7, 15, 30, 60, and 90 consecutive days. Thus, our design simulates early (≤ 15 days), intermediate (30–60 days) and prolonged (90 days) exposures, approximating progressive real-life use patterns in humans. A whole-body exposure system was used,^4^ with animals placed inside a closed glass chamber with a 4mm diameter orifice for inserting a silicon hose connected to an electric air machine and attached to the waterpipe device.^21^ A commercially available apple-flavored *moassel* tobacco (Al Nakhla Tobacco Company – Free Zone S.A.E^®^, Cairo, Egypt) was used together with 0.5% of non-washed tobacco and an instant lightening charcoal by Bamboo Brasil (Egitape Importação e Exportação. LTDA^®^, Florianópolis, Santa Catarina). Animals were exposed to one 2-second puff interspersed with 58 seconds of fresh air, totaling 30 minutes/day, which is the average length of one human smoking event, according to Hakim, et al.^22^ (2011) (Figure 1). This protocol was selected because it is similar to human breath topography during waterpipe use.^23^ As mice are known to perform grooming as a hygiene method, the whole body exposure system allows the deposition of chemical compounds on mice tongue from the smoke particles present on their fur. The control group was exposed only to air under the same conditions of the experimental groups and euthanized at 90 days. The other groups were euthanized at 7, 15, 30, 60, and 90 days.

### Tongue microscopic analysis

The mice tongues were surgically removed after euthanasia, fixed with 10% formaldehyde, and included in paraffin. For the immunohistochemical study, three micrometers sections were dewaxed, rehydrated and treated in 6% H_2_O_2_ methanol solution (1:1) for 30 minutes to stop endogenous peroxidase activity. For antigen retrieval, the sections were treated with citrate buffer pH 6.0 in a water bath at 96°C for 40 minutes. Non-specific binding sites were blocked with 5% skim powdered milk with phosphate-buffered saline (PBS) solution for 40 minutes. The sections were then incubated with mouse monoclonal antibody against DNMT1 (60B1220.1, 1:1500 dilution, Novus Biologicals, Centennial, EUA) and DNMT3b (NB300-516, 1:500 dilution, Novus Biologicals, Centennial, EUA) at 4°C overnight. Incubation with EnVision^TM^(DAKO North America Inc., Carpinteria, EUA) and DAKO Liquid DAB + Substrate Chromogen System^TM^ (3,3’-dyaminobenzyne) (DAKO North America Inc., Carpinteria, EUA) was performed for antigen-antibody complex visualization, followed by counterstaining with Harris hematoxylin. As negative control, the primary antibodies were omitted from the reaction sequence. As positive control, we used placental tissue specimens.^16^

Nuclear immunopositivity for DNMT1 and DNMT3b in epithelial cells was quantified. To ensure the reliability of the measurements, one calibrated, blinded observer evaluated the images. Intra-observer reliability was assessed by randomly selecting a subset of images and repeating the cell counting 15 days later (Intraclass Correlation Coefficient p>0.8). In each sample, immunopositive cells were counted in four equidistant fields per tongue site (dorsal surface, ventral surface, and lateral borders) using ImageJ version 1.51p (National Institutes of Health, Bethesda, Maryland, USA). Counting was performed at 400x magnification, with images captured using a light microscope (Axiostar Plus, Carl Zeiss, Oberkochen, Germany). This procedure was adapted from the methods described by Daniel, et al.^16^ (2016), Chrun, et al.^24^ (2017), and Wu, et al.^25^ (2018). The final outcome was determined by calculating the ratio of immunopositive cells to the total number of cells for each tongue site.

For inflammation cells analysis, three-micrometers sections were obtained and stained with hematoxylin and eosin (H&E). Inflammatory cells (polymorphonuclear and mononuclear cells) were quantified using the software *ImageJ* 1.51p version (Health National Institute, Bethesda, Maryland, EUA), according to Bósio et al.^27^ (2014) and classified as absent (0-10 cells/area), mild (11-25 cells/area), moderate (26-65 cells/area) and severe inflammation (more than 65 cells/area).^26^ Evaluation was conducted by one blinded and calibrated evaluator (p>0.8) as previously described.

### Statistical analysis

Data were analyzed in SPSS^®^ software version 11 (SPSS Inc., Headquarters, EUA). Fisher’s exact test was used to classify inflammatory infiltrate. Kruskal-Wallis test analyzed the incidence of nuclear immunopositivity for each DNMT. Pairwise comparisons were made using the Dunn-Bonferroni test. For all tests, differences were considered significant when p<0.05.

## Results

**Figure 2:**
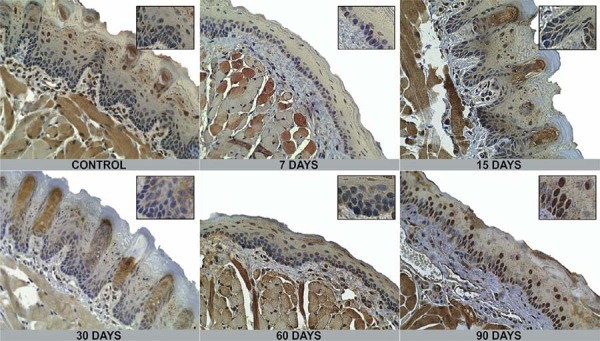
Epithelial immunohistochemical expression of DNMT1 at each exposure time. Main images were magnified at 100x and zoomed sub-image at 400x. Epithelial cells are stained in the nuclei in brown according to exposure period.

**Figure 3:**
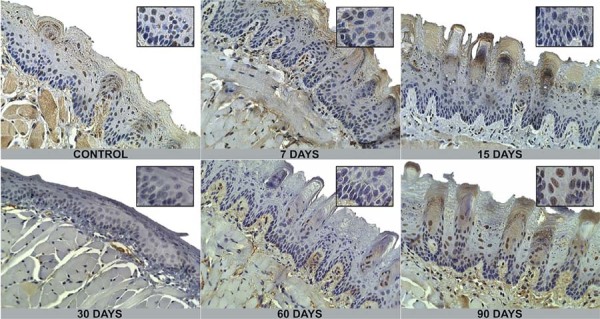
Epithelial immunohistochemical expression of DNMT3b at each exposure time (400x). Main images were magnified at 100x and zoomed sub-image at 400x. Epithelial cells are stained in the nuclei in brown according to exposure period.

**Figure 4:**
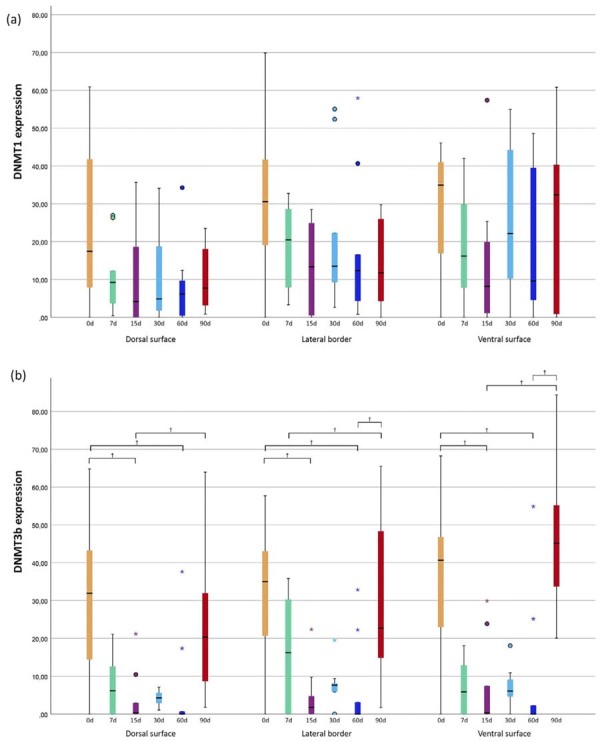
Boxplots showing enzyme expression pattern according to exposure times (in days) on each tongue site for DNMT1 (a) and DNMT3b (b). The graphic presents the variable distribution of our data in a five number summary (minimum, first quartile, mean, upper quartile and maximum); circles=outliers; *=extreme outliers; †=statistically significant difference.

Immunohistochemical expression of DNMT1 and DNMT3b was localized in the nuclei of epithelial cells (Figure 2 and Figure 3). The percentage of positive cells for DNMT1, as shown in Table 1 and Figure 4, showed no statistically significant differences when compared with exposure time or tongue site. DNMT3b immunopositivity showed statistical differences according to exposure time and tongue site, as shown in Table 2 and Figure 4.

**Table 1:** DNMT1 immunopositivity percentage according to waterpipe exposure times on tongue sites.

		DORSAL SURFACE	VENTRAL SURFACE	LATERAL BORDER	P value
Exposure Times	Control	17.45 (36.25)	34.95 (25.52)	30.55 (23.25)	0.671
	7 days	9.22 (12.49)	16.20 (23.79)	20.48 (22.37)	0.264
	15 days	4.15 (19.31)	8.21 (20.46)	13.32 (25.31)	0.774
	30 days	4.85 (20.90)	22.09 (42.25)	13.51 (30.15)	0.197
	60 days	6.12 (10.07)	9.61 (37.69)	12.33 (19.04)	0.217
	90 days	7.74 (15.24)	32.37 (41.60)	11.76 (22.83)	0.255
P-value		0.337	0.385	0.169	

Data shown as median (interquartile range).

**Table 2:** DNMT3b immunopositivity percentage according to waterpipe exposure times on tongue sites.

		DORSAL SURFACE	VENTRAL SURFACE	LATERAL BORDER	P value
Exposure Times	Control	31.95 (31.89)AB*	40.68 (31.41)AB	35.03 (28.03)AB*	0.005
	7 days	6.16 (13.57)*#	5.84 (13.47)C*	16.23 (30.53)#	0.004
	15 days	0.34 (4.80)AC*	0.31 (11.53)A	1.76 (5.97)AC*	0.009
	30 days	4.28 (4.03)*	6.08 (5.95)	7.62 (2.90)*	0.024
	60 days	0.00 (4.83)B*	0.00 (7.98)BD	0.00 (7.92)BD*	0.008
	90 days	20.33 (27.96)C*	45.16 (22.68)CD	22.74 (35.12)CD*	0.006
P-value		0.000	0.000	0.000	

Data shown as median (interquartile range).

Same letters indicate statistical differences between rows in the same column.

Same symbols indicate statistical differences between columns in the same row.

Polymorphonuclear and mononuclear cells quantification showed no statistically significant differences between the groups, with most cases classified as absent inflammation. Mild inflammation occurred in only 10% of the control group (when analyzing dorsal surfaces) and in the 90-day group (when analyzing dorsal and lateral border surfaces), whereas 15- and 30-day groups represented up to 22% of cases with mild inflammation on dorsal surfaces. Ventral surfaces exhibited absent inflammation in all study groups.

**Figure 5:**
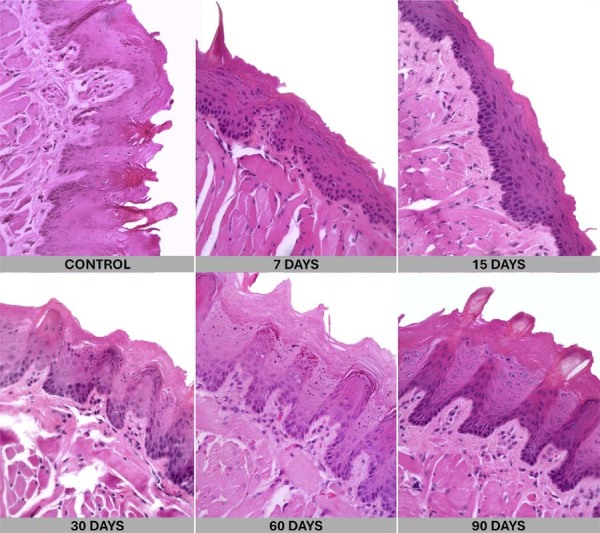
Representative histological (H&E) images of the dorsal surface in control and exposed groups (7, 15, 30, 60, and 90 days of waterpipe smoke exposure).

Analysis found no macroscopic alterations on the animals’ oral mucosa in any of the experimental groups. Representative hematoxylin and eosin (H&E) images from all experimental groups are presented in Figure 5 to illustrate the epithelial architecture and inflammatory pattern observed across exposure times. Consistent with the quantitative analysis, the microscopic evaluation revealed no significant signs of acute or chronic inflammation. Although mild inflammatory infiltrates were occasionally observed in some samples, these findings lacked statistical significance when comparing the groups. Overall epithelial morphology remained within normal histological limits and, importantly, no signs of epithelial dysplasia were identified in any of the samples across all experimental groups.

## Discussion

Lack of information about the harmful effects of waterpipe smoke has contributed to its worldwide use. Some studies have associated the habit of waterpipe smoking with a higher risk of developing lung and head and neck cancer; however, few microscopic studies have analyzed waterpipe-related cell and DNA alterations.^6,27,28^ To the best of our knowledge, this is the first standardized study to evaluate the expression pattern of DNMT1 and DNMT3b and to assess inflammatory cell infiltration on different tongue sites of mice exposed to waterpipe smoke for different periods.

Concerning DNMT3b immunopositivity, analysis revealed statistically significant differences among the groups according to exposure time and tongue site, as shown in Table 2 and Figure 4. However, the 7, 15, 30, and 60day groups exhibited a similar expression pattern, all presenting lower values than the control group. Although it is well established that DNMT3b is responsible for de novo methylation, Gagliardi, Strazzullo and Matarazzo^29^ (2018) reported that DNMT3b also works in conjunction with DNMT1 in DNA methylation maintenance during cell division. When these two enzymes present lower expression (as found here), DNA hypomethylation (global hypomethylation)^30^ may occur. It is strongly linked to chromosomal instability and proto-oncogenes activation, both of which are frequently reported as a common occurrence in early carcinogenesis.^28,30^ Moreover, few proto-oncogenes inactivation depends exclusively on DNMT3b activity, such as CREB, FOS, SP1, SP4, C/EBPα, and NF-κB p65.^31^ Thus, lower expression of DNMT3b in the groups at 7, 15, 30, and 60 days suggests that waterpipe smoke may promote proto-oncogenes activation, favoring early phases of carcinogenesis. Based on all these possibilities, we can expect a possible DNA hypomethylation pattern (both global and proto-oncogenes promoter regions) caused by initial periods of waterpipe smoke exposure.

Contrasting with these results, the 90-day group showed increased DNMT3b expression, in a level similar to that of the control group. Although the mice were not exposed beyond 90 days, more time of exposure could have resulted in higher expressions of DNMT3b and thus a tendency for DNA hypermethylation. Despite no evidence in the literature relating to DNMT3b down-regulation followed by up-regulation in oral cancer, this pattern has been described in lymphomas, with initial hypomethylation leading to proto-oncogenes activation and chromosomal instability, followed by secondary hypermethylation, which may silence important tumor suppressor genes.^32,33^ When this possible occurrence is associated with OSCC, and DNMT3b is overexpressed, the latter is commonly associated with lymph node involvement, tumor recurrence and a poor prognosis.^17^ Moreover, other clinical and histological consequences of the tumor may differ according to the tumor suppressor genes that are silenced by the hypermethylation process.^18,34,35^ In the 90-day group, the ventral surface showed DNMT3b higher expression when compared with control, and although there was no statistical significance with the ventral and lateral surfaces, this expression may show a higher tendency for oral cancer to develop on the ventral surface, as previously reported for OSCC in cigarette smokers.^5,28^ This higher frequency of oral cancer on the ventral surface may be associated with its non-keratinized epithelium, which is less protective and more vulnerable to several products originated from the tobacco burning process^9,36,37^ which are also found in waterpipe smoke, such as nicotine, tar, polycyclic aromatic hydrocarbons, volatile aldehydes, phenols, carbon monoxide and heavy metals.^5^ Even though the same compounds are found in tobacco and waterpipes, the latter present a higher concentration of them, with levels corresponding up to 10 cigarettes per waterpipe smoking session.^38,39^ However, the amounts of toxicants are still uncertain owing to variations in time and days of use.^7,9,40^

Regarding DNMT1, despite no statistical difference found for any group exposed to waterpipe smoke, all groups showed a lower expression on the dorsal surface and the lateral border when compared with control. On the ventral surface, as in DNMT3b, there was low expression from 7 to 60 days and an increase similar to control at 90 days. Lower expression of both DNMT1 and DNMT3b may lead to DNA hypomethylation, thus favoring initial carcinogenesis. Moreover, in all exposure periods waterpipe smoke was unable to induce the development of acute or chronic inflammation cells studied in the mice’s tongues.

Some study limitations should be acknowledged. Enzyme activity and specific DNA methylation patterns at promoter regions were not directly assessed, thus the conclusions regarding hypo or hypermethylation are based on DNMT1 and DNMT3b immunoexpression rather than on functional assays. Moreover, the experiment employed a whole-body exposure model which simulates real-life exposure to waterpipe smoke but does not isolate the effects of direct inhalation on oral tissues. Additionally, the sample was limited to female Swiss mice, which may restrict generalizing the results. Future studies combining genome-wide methylation analysis with longer exposure periods are needed to confirm these findings and clarify the molecular mechanisms linking waterpipe smoke exposure to DNA methylation disturbances and oral carcinogenesis.

Although this research did not evaluate enzyme activity or DNA methylation pattern, waterpipe smoke decreased DNMT3b and DNMT1 expression in short periods of exposure in mice, contributing to a possible hypomethylation status on all tongue sites, with ventral surface showing normal regulation or even upregulation of DNMT3b after a 90-day exposure. We cannot discard a possible DNA hypermethylation event caused by longer periods of waterpipe smoke exposure. We reinforce that further studies are needed to improve our knowledge about the effects of waterpipe smoke on the overt epigenetic status.
